# Myocardial Perfusion Reserve but not fibrosis predicts outcomes in initially asymptomatic patients with moderate to severe aortic stenosis: the PRognostic Importance of MIcrovascular Dysfunction in AS study- PRIMID AS

**DOI:** 10.1186/1532-429X-18-S1-O36

**Published:** 2016-01-27

**Authors:** Anvesha Singh, Michael Jerosch-Herold, John P Greenwood, Colin Berry, Dana K Dawson, Chim C Lang, Damian J Kelly, David Sprigings, Jeffrey P Khoo, Kai Hogrefe, Richard P Steeds, Vijay Anand Dhakshinamurthy, Gerry P McCann

**Affiliations:** 1 Cardiovascular Sciences, University of Leicester and the NIHR Leicester Cardiovascular Biomedical Research Unit, Leicester, UK; 2 Multidisciplinary Cardiovascular Research Centre & The Division of Cardiovascular and Diabetes Research, Leeds Institute of Genetics, Health & Therapeutics, Leeds University, University of Leeds, Leeds, UK; 3 BHF Glasgow Cardiovascular Research Centre, University of Glasgow, Glasgow, UK; 4 Cardiovascular Medicine Research Unit, University of Aberdeen, Aberdeen, UK; 5 Division of Cardiovascular and Diabetes Medicine, Ninewells Hospital and Medical School, Dundee, UK; 6 Cardiology, Royal Derby Hospital, Derby, UK; 7 Cardiology, Northampton General Hospital, Northampton, UK; 8 Cardiology, Grantham and District Hospital, Grantham, UK; 9 Cardiology, Kettering General Hospital Foundation Trust, Kettering, UK; 10 Cardiovascular Medicine, Queen Elizabeth Hospital, Birmingham, UK; 11 Cardiology, University Hospital, Coventry, UK; 12 Brigham & Women's Hospital, Boston, MA USA

## Background

The timing of surgery in asymptomatic patients with aortic stenosis (AS) is controversial. Adverse LV remodeling is related to prognosis in AS. Cardiovascular magnetic resonance (CMR) can identify diffuse and focal myocardial fibrosis, by T1 mapping and late gadolinium enhancement (LGE) respectively, as well as myocardial perfusion reserve (MPR), which is inversely related to symptoms and an independent predictor of exercise capacity in severe AS. The aim of this study was to determine whether MPR and other CMR markers of LV remodeling are of prognostic value in asymptomatic AS.

## Methods

Asymptomatic patients with moderate to severe AS and matched healthy controls were recruited in this prospective, multi-centre study, and underwent echocardiography and a stress CMR at 3T. CMR analysis was blinded and undertaken in a core lab. Investigations were not reported unless there was a clinical indication. Patients were followed for 12-30 months and outcomes were adjudicated by 2 independent Cardiologists, blinded to test results. Clinical outcome was a composite of: the development of typical symptoms, major adverse cardiovascular events or aortic valve replacement over a median follow-up of 18 months.

## Results

174 patients and 23 controls were recruited. Compared to controls, LV volumes, mass, MPR and LGE were significantly different in patients, but there was no significant difference in ECV. 60 patients had a clinical event during follow-up (34.5%). There was no difference in comorbidities between those with and without an outcome. After adjusting for sex: AS severity, MPR, valvulo-arterial impedance and LV mass/volume were univariate predictors of the outcome. CMR measures of fibrosis (LGE, native T1 and ECV) did not predict outcome. On stepwise multivariate analysis, severe AS (HR 0.17 (0.07-0.43) p = 0.0002) and MPR (HR 0.60 (0.40-0.92) p = 0.0197) were independent predictors of outcome.

## Conclusions

MPR, but not markers of fibrosis, is a predictor of outcome in initially asymptomatic patients with AS. Further randomized trials are needed to determine whether MPR can improve outcomes in asymptomatic AS.

**Table 1:** Demographic, echocardiographic and CMR data for patients and controls

	AS Patients (n = 174)	Healthy Controls (n = 23)	p-value
Age (years)	66.2 ± 13.3	68.3 ± 8.8	0.331
Male (n (%))	133 (76.4)	16 (69.6)	0.471
Echocardiography data			
AV Vmax (m/s)	3.86 ± 0.56	1.35 ± 0.27	<0.001*
AVAI (cm2/m2)	0.57 ± 0.14	1.71 ± 0.36	<0.001*
Lateral E/e'	9.88 ± 3.72	8.07 ± 2.97	0.026*
VAI (Echo) (mmHg/ml/m2)	3.96 ± 1.06	3.67 ± 0.76	0.220
CMR data			
LVEDVI (ml/m2)	87.58 ± 18.27	78.16 ± 9.40	<0.001*
LVESVI (ml/m2)	38.28 ± 10.65	32.11 ± 5.03	<0.001*
LVEF (%)	56.7 ± 4.95	58.9 ± 3.67	0.044*
LVMI (g/m2)	57.69 ± 13.85	44.31 ± 7.20	<0.001*
LV mass/volume (g/ml)	0.66 ± 0.11	0.57 ± 0.08	<0.001*
VAI (MRI) (mmHg/ml/m2)	3.81 ± 0.82	3.50 ± 0.74	0.078
Global MPR	2.27 ± 0.70	3.16 ± 0.65	<0.001*
LGE present (n,%)	82 (47.1)	5 (21.7)	0.025*
% LGE (%)	4.20 ± 3.76	2.00 ± 2.21	<0.001*
Native myocardial T1 (ms)	1131.9 ± 69.54	1092.3 ± 34.29	<0.001*
ECV (%)	24.82 ± 2.43	25.05 ± 2.57	0.680

AV Vmax=peak aortic jet velocity, AVAI=aortic valve area indexed to BSA, VAI=valvulo-arterial impedance, LVEDVI=left ventricular end-diastolic volume indexed to BSA, LVESVI=left ventricular end systolic volume indexed to BSA, LVEF=left ventricular ejection fraction, LVMI=left ventricular mass indexed to BSA, MPR=myocardial perfusion reserve, LGE=late gadolinium enhancement, ECV=extracellular volume fraction

